# Trends in diabetes mellitus among Taiwanese adolescents and young adults during 2000-2009: a national population-based cohort study

**DOI:** 10.1186/1687-9856-2013-S1-P31

**Published:** 2013-10-03

**Authors:** Meng-Che Tsai, Chih-Ching Chang, Yen-Yin Chou, Shio-Jean Lin, Sheng-Hsiang Lin

**Affiliations:** 1 Department of Pediatrics, National Cheng Kung University, College of Medicine, National Cheng Kung University, Tainan, Taiwan; 2 Department of Environmental and Occupational Health, College of Medicine, National Cheng Kung University, Tainan, Taiwan; 3 Research Center of Clinical Medicine, Cheng Kung University Hospital, College of Medicine, National Cheng Kung University, Tainan, Taiwan; 4 Institute of Clinical Medicine, College of Medicine, National Cheng Kung University, Tainan, Taiwan

## Aims

To describe trends in the prevalence and incidence of diagnosed diabetes mellitus (DM) among Taiwanese adolescents and young adults.

## Methods

A subset of Taiwan National Health Insurance Research Database containing complete inpatient and outpatient data of one million beneficiaries randomly drawn from the national population of 23 millions was used for this retrospective longitudinal study during 2000-2009. Patients aged 10-24 years old who had at least two outpatient visit claims and/or one inpatient hospitalization claim for diabetes based on the International Classification of Disease, 9^th^ Revision, Clinical Modification (ICD-9-CM) code 250.XX were included. Patients were further subdivided as having type 1 DM, if they had been hospitalized due to diabetic ketoacidosis (ICD-9-CM code 250.1X) and/or had received claims for ICD-9-CM 250.X1 or 250.X3. Those who failed abovementioned criteria for type 1 DM were classified as having type 2 DM. Age-specific and age-sex-adjusted standardized annual incidence and prevalence with 95% confidence interval (CI) by the calendar year 2004 were calculated to describe their trends in different gender and age groups.

## Results

During the study period, the age-sex-adjusted prevalence and mean annual incidence of diagnosed type 1 DM were 521.59 per 100,000 enrollees (95% CI 514.89-528.59) and 3.24 per 100,000 enrollees (95% CI 2.71-3.77), respectively. No remarkable sexual difference in the annual incidence of type 1 DM was noted, whereas a male/female ratio of 0.85 (95% CI 0.82-0.88) in the prevalence was observed. The annual incidence of type 1 DM decreased with age and remained stable over these 10 years; while the prevalence remained constant through adolescence and varied from year to year. Meanwhile, the adjusted prevalence and mean annual incidence of type 2 DM were 834.14/100,000 (95% CI 825.64-842.63) and 120.19/100,000 (95% CI 116.96-123.43), respectively. No remarkable sexual predominance in the prevalence of type 2 DM was noted, whereas a male/female ratio of 0.86 (95% CI 0.79-0.92) was observed in the prevalence. The annual incidence of type 2 DM increased with age and decreased gradually over the recent 10 years; while the prevalence was still on the increase in this age group.

## Conclusion

The incidence and prevalence of type 2 DM came to outnumber that of type 1 DM during adolescence. Although the incidence of newly diagnosed type 2 DM decreased, a rising trend in the prevalence of type 2 DM still existed. A public health policy may be needed to combat the emerging health issue of adolescent diabetes.

